# Strategies for reducing per‐sample costs in target capture sequencing for phylogenomics and population genomics in plants

**DOI:** 10.1002/aps3.11337

**Published:** 2020-04-14

**Authors:** Haley Hale, Elliot M. Gardner, Juan Viruel, Lisa Pokorny, Matthew G. Johnson

**Affiliations:** ^1^ Department of Biological Sciences Texas Tech University Lubbock Texas 79409 USA; ^2^ The Morton Arboretum Lisle Illinois 60532 USA; ^3^ Department of Biology Case Western Reserve University Cleveland Ohio 44106 USA; ^4^ Singapore Botanic Gardens National Parks Board 1 Cluny Road 259569 Singapore; ^5^ Royal Botanic Gardens Kew, Richmond Surrey TW9 3DS United Kingdom; ^6^ Present address: Centre for Plant Biotechnology and Genomics (CBGP) UPM‐INIA 28223 Pozuelo de Alarcón (Madrid) Spain

**Keywords:** enzymatic fragmentation, herbariomics, high‐throughput workflow implementation, Hyb‐Seq, low‐cost sequence capture, pooling and multiplexing strategies

## Abstract

The reduced cost of high‐throughput sequencing and the development of gene sets with wide phylogenetic applicability has led to the rise of sequence capture methods as a plausible platform for both phylogenomics and population genomics in plants. An important consideration in large targeted sequencing projects is the per‐sample cost, which can be inflated when using off‐the‐shelf kits or reagents not purchased in bulk. Here, we discuss methods to reduce per‐sample costs in high‐throughput targeted sequencing projects. We review the minimal equipment and consumable requirements for targeted sequencing while comparing several alternatives to reduce bulk costs in DNA extraction, library preparation, target enrichment, and sequencing. We consider how each of the workflow alterations may be affected by DNA quality (e.g., fresh vs. herbarium tissue), genome size, and the phylogenetic scale of the project. We provide a cost calculator for researchers considering targeted sequencing to use when designing projects, and identify challenges for future development of low‐cost sequencing in non‐model plant systems.

Target sequence capture consists of enriching genomic libraries for regions of interest (nuclear or organellar), such as highly conserved regions (e.g., ultra‐conserved elements, Faircloth et al., 2012; or anchors, Lemmon et al., 2012), more variable low‐copy orthologous loci (e.g., exons plus their flanking non‐coding introns, Mandel et al., 2014; Weitemier et al., 2014), or functional genes (Gardner et al., 2016; Moore et al., 2018). In land plants, the Hyb‐Seq method has recently become a standard procedure for generating large amounts of sequence data for phylogenomics of non‐model organisms (Crowl et al., 2017; Gernandt et al., 2018; Stubbs et al., 2018; Medina et al., 2019). In addition to the resulting wealth of data, other advantages of this approach are the low levels of missing data (minimizing issues with orthology) and its cost‐effectiveness (allowing for broad taxon sampling) (McKain et al., 2018; Dodsworth et al., 2019).

The most common design for target sequence capture in plants involves developing probes that target low‐copy orthologous loci from a narrow set of in‐group taxa (Gardner et al., 2016; Vatanparast et al., 2018; Villaverde et al., 2018; Soto Gomez et al., 2019). Alternatively, universal gene sets that work across a wider variety of taxa have also been developed; for example, in Compositae (Mandel et al., 2014), Bryophyta (Liu et al., 2019), ferns (Wolf et al., 2018), or flowering plants (Buddenhagen et al., 2016; Johnson et al., 2018). As the use of target capture has expanded, so has its applicability, it being used at both interspecific and infraspecific levels (Villaverde et al., 2018; Murphy et al., 2020), and even to estimate ploidy (Viruel et al., 2019).

Here, we describe approaches for target capture sequencing that reduce per‐sample costs at each stage along the target sequence‐capture wet‐lab pipeline: (1) DNA extraction, (2) library preparation, (3) hybrid enrichment, and (4) sequencing; including pooling strategies for stages 1–3, and required quality controls in between stages (Fig. 1). Each strategy will have implications not only for laboratories desiring high throughput, but also for the broad adoption of targeted sequencing in laboratories with limited resources. Taken together, the cost‐saving modifications may reduce the overall per‐sample cost by 70% or more compared with service providers, and by 50% or more compared with standard in‐house laboratory procedures (Table 1).

**Figure 1:**
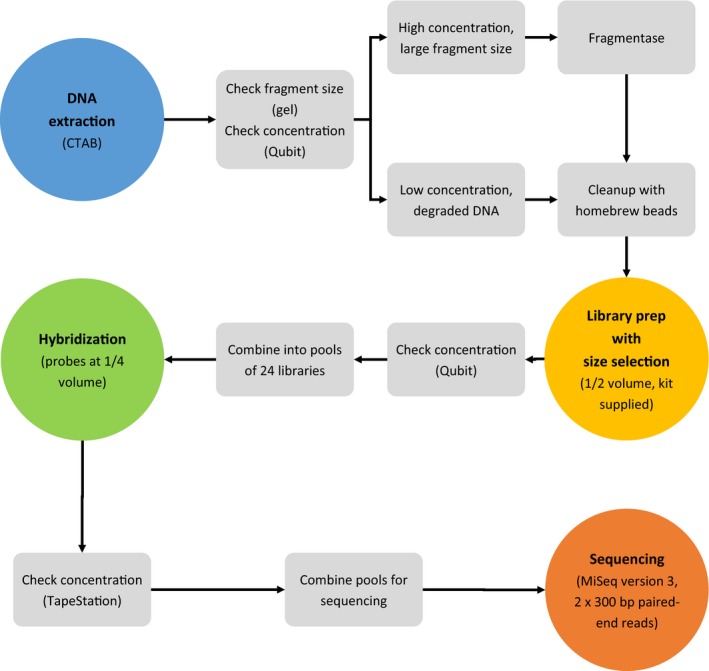
Example workflow diagram for cost‐effective target capture sequencing. This workflow is designed to be used by a research group trying targeted sequencing for the first time on a set of 96 samples and represents a 50% reduction in reagent and consumable costs compared with traditional methods (see text). The equipment required for this workflow is a centrifuge capable of 13,000 *g*, micropipettes (preferably multichannel), −80°C and −20°C freezers, a water bath, a thermocycler, a magnetic bead separator, a Qubit fluorometer (Thermo Fisher Scientific, Waltham, Massachusetts, USA), and access to a method of hybridization pool quantification (e.g., TapeStation [Agilent Technologies, Santa Clara, California, USA], BioAnalyzer [Agilent Technologies], qPCR).

**Table 1:** Comparison of consumable costs between conventional and low‐cost methods in the target capture workflow. See text for full explanation of methods and alternatives and for equipment cost considerations.^a^

Hyb‐Seq procedure	Usual technique	Cost‐saving technique	Usual technique price (per sample)	Cost‐saving technique price (per sample)	Estimated fold‐cost savings
DNA extraction	QIAGEN DNeasy 96	CTAB	$3.11	$0.29	10.72
Quality control	TapeStation	Qubit + gel	$3.21	$1.30	2.47
Fragmentation	Sonicator	Fragmentase	$6.76	$1.41	4.79
Library prep	NEBNext Ultra II DNA full volume	NEB half volume	$29.20	$14.60	2.00
Purification	AMPure beads	Homebrew beads	$2.26	$0.08	28.25
myBaits	24‐plex	24‐plex + dilute myBaits	$2.16	$0.56	3.86
Sequencing	MiSeq 2 × 300 bp (96‐plex)	HiSeq X (384‐plex)	$18.50	$4.42	4.19
**Overall**			**$65.20**	**$22.66**	**2.88**

a Prices (in U.S. dollars) are approximate and may vary by institution.

Our suggestions assume that staff time from existing employees is included. Depending on the scale of the project, users can consider using an external service for some or all of these processes, but service providers may not modify the protocols to reduce costs following our guidelines. For example, DNA extractions may be done by a third party, although not all facilities are experienced in dealing with the secondary compounds that often hinder extraction from some plant tissues. Many sequencing facilities offer library preparation services; however, because target capture occurs between library preparation and sequencing, facilities may not be equipped for all parts of the workflow. If researchers choose an external service, special permits might be needed when sending DNA or materials from certain taxa (e.g., to comply with the Convention on International Trade in Endangered Species of Wild Fauna and Flora [CITES]). With the suggestions and modifications outlined here, we believe targeted sequencing is feasible for any research group with access to standard laboratory equipment at a reasonable per‐sample cost considering the amount of data generated.

## DNA EXTRACTION

Initial DNA template quality has perhaps the highest impact on final quality of sequence data. A variety of existing extraction protocols account for tissue type (e.g., leaves, petioles, stems, roots), age of the specimen (recent vs. old), preservation strategy (e.g., silica vs. air dried, doused in ethanol, mercuric chloride biocide treatment), and other attributes impacting template quality and output quantity (Brewer et al., 2019; Forrest et al., 2019; Shapiro et al., 2019; Andermann et al., 2020). Plant cells can have up to three wall layers that need to be crushed prior to DNA purification. There is a wide variety of tissue lysis equipment available; the choice depends on budget, consumables (i.e., tubes), workflow, and the tissue itself. On the high end, samples can be processed in tubes with automated ball mills, such as CryoMill (Retsch, Haan, Germany), or tissue homogenizers, including FastPrep24 (MP Biomedicals, Irvine, California, USA) and Geno/Grinder (SPEX SamplePrep, Metuchen, New Jersey, USA). The Geno/Grinder can also be used to process samples, in 24–384 tubes or in deep‐well titer plates. On the low end, it is possible to modify a reciprocating saw for use as a tissue grinder (Alexander et al., 2006; Dean et al., 2018) for a fraction of the cost of commercially available machines. The grinding media used for the ball mills can vary in size and material, allowing some modifiability for sufficient grinding of a variety of tissue types. For small numbers of samples, or for tissue that proves hard to process by automated means, grinding by hand is still effective. In addition to the classic porcelain mortar and pestle, autoclavable plastic pestles can also be used with standard microcentrifuge tubes (e.g., Pellet Pestle, DWK Life Sciences, Millville, New Jersey, USA). These plastic pestles work best with tissue that is thin and brittle or softened via soaking. Additional grinding power can be obtained by loading the pestle into a drill (Dean et al., 2018). However, it is recommended to grind tough and bulky tissue, such as extremely fibrous leaves or stems and thick or woody roots, with porcelain mortars and pestles while pouring liquid nitrogen directly onto the tissue.

The simplest ways to grind fresh tissue are to dry it on silica gel and then pulverize it using ball bearings or to grind it with a lysis buffer (e.g., cetyltrimethylammonium bromide [CTAB]), either using a mortar and pestle (Drábková et al., 2002) or a hand‐operated homogenizer in a 2‐mL tube (Ahmed et al., 2009). However, grinding fresh tissue is often facilitated by freezing the tissue with dry ice or liquid nitrogen (Rogers and Bendich, 1988). In the absence of liquid nitrogen or dry ice, materials can be frozen at −80°C (or −20°C, depending on freezer availability) and ground in a pre‐chilled mortar and pestle (Sahu et al., 2012).

Following tissue breakup, a variety of commercial DNA purification kits are available (e.g., DNeasy Plant Mini Kit [QIAGEN, Hilden, Germany], Zymo Quick DNA Plant Kit [Zymo Research, Irvine, California, USA], innuPREP Plant DNA Kit [Analytik Jena, Jena, Germany], GenElute Plant Genomic DNA Miniprep Kit [Sigma‐Aldrich, St. Louis, Missouri, USA], among many others), which represent a tradeoff between convenience and cost. These kits usually include nearly all reagents needed (except for laboratory basics such as ethanol or isopropanol)—allowing for a streamlined timeline—but do come at a steeper price. Although commercial protocols are not easily modified, they are optimized to reduce toxic emissions and therefore do not typically require a fume hood.

Customizable DNA extraction protocols, such as the CTAB method (Doyle and Doyle, 1987), have been used in plants for decades and are as effective as kits at recovering enough DNA for high‐throughput sequencing, even from 50–250‐year‐old herbarium specimens (Brewer et al., 2019; Viruel et al., 2019). For old herbarium material, CTAB extraction will often produce a much higher DNA yield than a kit, although the DNA may contain more impurities and therefore require cleanup. Chemicals and solutions used in the standard CTAB extraction protocol are simple to make and inexpensive, as they are often purchased in bulk. Once available, samples can be processed in large or small numbers with no change in per‐sample cost. The CTAB protocol can be easily modified to suit different tissue types and ages, regardless of preservation strategies, and upscaled to process 96–192 samples at a time (see Dryad repository associated with Beck et al., 2012a, b; and supplementary materials for Larridon et al., 2020) with a Geno/Grinder. For dried and old herbarium materials, higher concentrations of β‐mercaptoethanol (i.e., 0.4%) are recommended in the CTAB step, a fast vortex and clean‐up with chloroform : isoamyl alcohol, and long incubations in isopropanol at −20°C (e.g., 48 h, or even up to a week for precious and difficult materials; Larridon et al., 2020). Phenol cleaning is highly recommended in plant DNA isolation protocols (Drábková et al., 2002), but high DNA concentrations can be achieved without it. Extra modifications (recommended when polysaccharides and secondary metabolites are abundant) include sorbitol and high‐salt (4 M NaCl) CTAB (e.g., for mucilage‐rich tissue, Tel‐Zur et al., 1999; Štorchová et al., 2000) or 2% polyvinylpyrrolidone (PVP) (e.g., for latex‐rich tissue, Michiels et al., 2003). When phenol use is restricted (e.g., in light of health and safety concerns), additional clean‐up steps are recommended, such as column (e.g., CsCl‐EtBr density gradient centrifugation, Albach and Chase, 2001; Ahmed et al., 2009; Höpke et al., 2019) or bead cleaning (e.g., solid‐phase reversible immobilization [e.g., SPRIselect beads, Beckman Coulter, Indianapolis, Indiana, USA]; Shee et al., 2020), and even additional modified CTAB protocols optimized for plant lineages with high levels of polysaccharides and secondary metabolites (Štorchová et al., 2000; Sahu et al., 2012).

An input of 200 ng of DNA is commonly recommended for genomic library preparation (e.g., see the manuals for NEBNext Ultra II DNA [New England Biolabs, Ipswich, Massachusetts, USA], TruSeq Nano DNA LT/HT [Illumina, San Diego, California, USA], or KAPA HTP/LTP library prep [Roche Sequencing Solutions, Pleasanton, California, USA] kits), which can be easily achieved for fresh or silica gel materials, and is highly probable for recently dried materials. Older herbarium specimens might need multiple parallel DNA isolations from the same sample to obtain 200 ng, and in some cases up to 1 μg may be required to overcome extremely degraded extractions, as non‐endogenous DNA will be more abundant in this latter case (Wales and Kistler, 2019). In practice, library prep protocols can construct genomic libraries from input amounts as low as 5 ng of DNA, even from herbarium specimens (Brewer et al., 2019), at the cost of potentially losing some fragment diversity (Johnson et al., 2018).

## FRAGMENTATION

For targeted sequencing, fragmentation of genomic DNA is likely necessary, unless working with degraded DNA (where the majority of fragments are already <500 bp after extraction). The appropriate average fragment size will ultimately depend on the desired sequence data—as will the library preparation kit and sequencing platform used later in the workflow—but fragment sizes between 350 and 550 bp are usually preferable (e.g., see NEBNext Ultra II DNA, TruSeq Nano DNA LT/HT, or KAPA HTP/LTP library prep kit manuals). There are two common DNA shearing methods, mechanical (nebulization or sonication) and enzymatic (with fragmentases/nickases), each with its own biases and errors. It is worth noting that enzymatic fragmentation does not have a significant effect on the read length, sequence quality, and percentage of sequences able to be aligned (Knierim et al., 2011).

**Figure 2:**
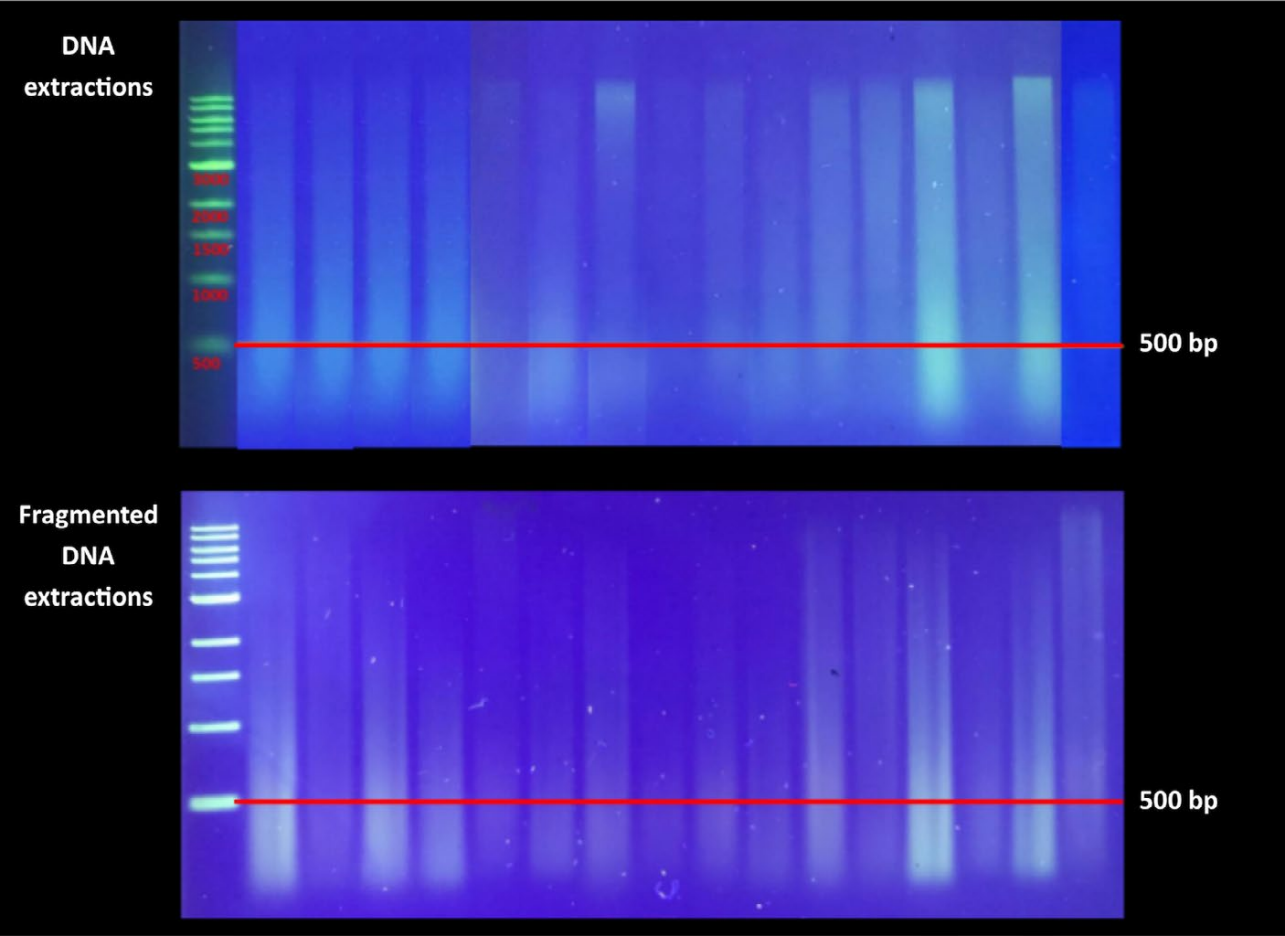
Effective enzymatic fragmentation of DNA extractions. Total genomic DNA eluted in 0.1 Tris‐EDTA was run on a 1% agarose gel at 180 V for 60 min and compared with the NEB 1‐kbp DNA ladder (New England Biolabs, Ipswich, Massachusetts, USA). Herbarium samples with a considerable concentration of DNA fragments longer than 500 bp (top) were digested with NEB Fragmentase (New England Biolabs) for 10 min, then run on a new gel (bottom) under the same conditions.

Mechanical methods of DNA fragmentation rely on adaptive cavitation technology, such as Bioruptor sonication devices (Diagenode, Liege, Belgium; e.g., Pico and Plus models), or use adaptive focused acoustics (AFA) technology, such as Covaris focused‐ultrasonicators (Covaris, Woburn, Massachusetts, USA), and have traditionally been seen as a reliable standard for high‐throughput sequencing (Meyer and Kircher, 2010). Mechanical fragmentation requires specialized equipment, software, and consumables that lead to an expensive start‐up cost and potential inefficiencies when working with large sample sizes. Some sonication devices are capable of processing 96+ samples at a time, such as Covaris’ L‐ and E‐series, but these machines are not widely available. Covaris shearing performance is independent of DNA concentration or base content (see https://covaris.com/wp-content/uploads/M020013.pdf, accessed 30 November 2019). We have successfully met our desired fragment sizes (e.g., 250–550 bp) starting from 25–55 μL DNA (in microTUBE‐50 AFA Fiber Screw‐Cap tubes [Covaris]) for a wide range of concentrations (i.e., 50–250 ng/μL), following manufacturer's protocols. Sonication is not recommended for shearing DNA already in <500‐bp fragments, as is frequently the case for herbarium specimens (Fig. 2); however, we have found that standard sonication protocols perform adequately even on partially degraded samples.

Enzymatic fragmentation is relatively new, yet considerably cheaper per sample, and it works with a much smaller starting template amount. For the NEBNext dsDNA Fragmentase kit (New England Biolabs), only 5 ng of starting DNA are needed, and the KAPA Frag Kit (Roche Sequencing Solutions) requires as little as 1 ng of starting DNA template. These kits can easily be used to create DNA fragments, ranging from 50 to 1000 bp, by shortening or extending incubation times. It is important to note that although optimal fragmentation conditions require incubation, fragmentase is still active at room temperature, so enzymatic fragmentation must be conducted quickly and efficiently. Other methods of enzymatic fragmentation (e.g., restriction digests used for RAD‐Seq) can lead to non‐random cuts that are not as desirable for targeted sequencing.

Using enzymatic fragmentation can drastically reduce costs relative to mechanical fragmentation; for example, NEBNext dsDNA Fragmentase represents a six‐fold cost savings in consumables compared with Covaris sonication (Table 1). Enzymatic fragmentation also eliminates the need for specialized equipment, requiring only regular microcentrifuge tubes and a heat block or water bath. We have found enzymatic fragmentation to be a good choice for high‐throughput targeted sequencing (Fig. 2), although the incubation time should be optimized for each plant lineage because remaining polysaccharides and secondary metabolites could have an impact. Library preparation kits that include enzymes for fragmentation as part of the protocol are conveniently available off the shelf (e.g., NEBNext Ultra II FS DNA and KAPA HyperPlus Kits) and are useful for researchers working with fresh or recently collected tissue. On the other hand, DNA template obtained from older herbarium specimens often does not need to be sheared; therefore, costs can be reduced for projects that include a high proportion of herbarium specimens by purchasing kits without fragmentase and then buying the enzyme module for the samples that require it. We note, however, that for partially degraded herbarium material in need of fragmentation, incubation times must be determined empirically, as slight increases in incubation time may lead to unusably small fragments. For these samples, mechanical fragmentation—which we have found to be more forgiving—can be simpler and more reliable if the relevant equipment is available (E. M. Gardner, M. G. Johnson, N. J. Wickett, and N. J. C. Zerega, unpublished data).

## QUALITY CONTROL

At several stages in the target enrichment workflow (e.g., following DNA isolation, post‐library preparation, to inform multiplexing/pooling), standard protocols require that quality (fragment size distribution) and quantity (concentration in ng/μL) of the input template be measured. There are many different methods of quality control that may be better for certain stages than others, depending on the level of certainty required in concentration, fragment size distribution, and molarity.

Accurate DNA quantification is essential prior to library preparation, when pooling samples for hybridization, and before sequencing. Quantitative PCR (qPCR) and droplet digital PCR (ddPCR) are industry standards, but the required reagents and equipment make their use not feasible for most phylogenomics or population genomics projects due to high per‐sample costs (Robin et al., 2016). Quantification of DNA using a fluorometer, such as the Qubit 4 (Thermo Fisher Scientific, Waltham, Massachusetts, USA) or the Quantus (Promega Corporation, Madison, Wisconsin, USA), is more cost effective and nearly as accurate (Simbolo et al., 2013). For this reason, fluorometric quantification—although not as accurate as ddPCR or qPCR (Robin et al., 2016)—has become the standard for large‐scale target enrichment projects.

Using single‐sample machines, such as the Qubit 4 or Quantus, can be time‐consuming in high‐throughput workflows, as the tube size does not allow for easy high‐throughput transfer of samples. However, with some creativity (e.g., using a deep‐well plate as a rack and dispensing reagents from a wide reservoir), tubes can be arranged to facilitate the use of multichannel pipettes, although samples must still be read one at a time. Careful pipetting and using enough sample (e.g., 2–3 μL) are essential for maintaining consistent accuracy. Some cost savings may be achieved by using alternative DNA‐binding reagents such as Quant‐iT PicoGreen (Thermo Fisher Scientific), AccuGreen, or AccuClear (Biotium, Fremont, California, USA), which are compatible with the Qubit 4. For very high‐throughput workflows, these assays can also be prepared on plates and analyzed with microplate readers (e.g., Infinite series [TECAN, Männedorf, Switzerland]).

Many researchers use the NanoDrop spectrophotometer (Thermo Fisher Scientific), which reports both DNA concentration and purity (i.e., A_260_/A_230_ and A_260_/A_280_ absorbance ratios) with minimal reagent costs after the initial equipment purchase. Concentrations reported by the NanoDrop are generally higher than those reported for the Qubit (Nakayama et al., 2016), especially for samples with impurities, and we therefore recommend it be used only for measuring the purity of DNA extractions (Simbolo et al., 2013). If no other option exists, the quantification of extractions using the NanoDrop may provide accurate estimates to proceed with library preparation, but we advise against using NanoDrop for quantification of prepared libraries.

For measuring fragment size, the most cost‐effective method is gel electrophoresis, which requires only the reagents to make the gel plus DNA‐binding dye, loading dye, and a DNA ladder of appropriate size. An agarose gel can be an effective method for assessing the fragment size distribution of eluted DNA in order to make sample‐specific decisions about fragmentation (Fig. 2). Low‐concentration DNA may not be visible on a gel, although increasing the dye concentration or post‐staining with additional DNA‐binding dye can sometimes overcome this problem. Protocols for casting gels with dye as well as for post‐staining can often be found in a manual or product information document created by the dye manufacturer.

Agilent's Fragment Analyzer, TapeStation, and Bioanalyzer systems (Agilent Technologies, Santa Clara, California, USA) are automated electrophoresis tools for quantitation of DNA concentration (ng/μL), fragment size distribution, and molarity. Advantages of these machines are extremely quick preparation and short runtimes, but equipment and reagent costs are expensive per sample. The Bioanalyzer system uses microfluidic technology on a specialized chip with built‐in channels. Although the Bioanalyzer system is more reliable and precise than the TapeStation (e.g., models 2200, 4150, and 4200), it was not built with high throughput in mind. Necessary equipment includes a benchtop instrument, a computer, a chip priming station, and an IKA vortex mixer (IKA Works, Wilmington, North Carolina, USA). Chips can only process up to 11 samples at a time and are not reusable if fewer than 11 samples are run.

In addition to the benchtop instrument itself, Agilent's TapeStation system also requires brand‐specific optical eight‐tube strips. The ScreenTapes used contain 16 assays for a range of DNA fragment sizes, and unused lanes can be re‐run later (within a few weeks), allowing for more flexibility than the Bioanalyzer. For higher throughput, the 4200 TapeStation system is compatible with brand‐specific 96‐well fully skirted optical plates. Processing takes around 1–2 min per sample, including data analysis, which is faster than the 30 min or more required for the Bioanalyzer. The Fragment Analyzer (systems 5200, 5300, and 5400) provides the highest throughput, although it requires the steepest initial expense. Fragment Analyzers process from 12 to 96 samples per tray, and trays can be loaded and programmed while a run is in progress.

The specialized equipment and consumables make using these Agilent systems undesirable, in terms of cost, for high‐throughput studies prior to library preparation or hybridization. Research groups piloting a high‐throughput workflow may want to use one of the Agilent systems as a spot check for low‐concentration libraries (i.e., <5 ng/μL), measured for concentration using their fluorometer of choice, along with a random sampling of a few medium‐ to high‐concentration libraries to judge the approximate molarity. Once comfortable with the target capture procedure, using only a fluorometer and agarose gel for quality control of DNA extractions and library preparations reduces per‐sample costs by two‐ or threefold. In contrast, the reliable reading of enriched library molarity prior to sequencing is still highly recommended (see below).

## PURIFICATION AND SIZE SELECTION

Purification of template is necessary many times throughout the target enrichment workflow, such as after DNA extraction, fragmentation, throughout library preparation, and following hybridization. SPRI beads are the most reliable and flexible method for purification in this context. SPRI beads are carboxyl‐coated paramagnetic beads that reversibly bind to DNA in the presence of polyethylene glycol (PEG) and salt. They can be used to purify samples by removing salts, enzymes, excess of primers, and adapter dimers. They can also be used for size selection of DNA fragments when preparing libraries.

Although commercially available SPRI beads can be expensive, which cautions against their use in high‐throughput studies, beads can be prepared in‐house at a much lower cost (Rohland and Reich, 2012). A detailed protocol for bead preparation can be found online (https://ethanomics.files.wordpress.com/2012/08/serapure_v2-2.pdf, accessed 30 September 2019), enabling the rapid and simple preparation of SPRI beads with comparatively more affordable reagents. Accuracy testing should be done immediately after bead preparation as well as after prolonged storage. These “homebrew” SPRI beads have been used with positive results in the elimination of very short fragments (e.g., primer‐dimer) and very large fragments (Rohland and Reich, 2012), for one‐tenth the price of commercially available Agencourt AMPure XP (Beckman Coulter) or other SPRI beads. Homebrew beads should be tested extensively before they are used for more specific size selection purposes (e.g., during library preparation). Notably, homebrew beads have been shown to be reliable for the many sample clean‐up steps throughout the target enrichment workflow (Rohland and Reich, 2012).

Although SPRI beads are cost efficient, some additional equipment and skills are crucial when they are used. Magnetic particle concentrators (MPCs) are required to pellet the paramagnetic beads during liquid transfer and removal steps. Different MPC versions are available, differing in size (0.2‐ and 1.5‐mL tubes/plates), shape, and placement of the magnets on the stand. These characteristics can make a huge difference in the ease and speed of use. Skills need to be developed to work with plates because beads can dry out quickly, resulting in a potentially large reduction of DNA or library yields (e.g., see NEBNext Ultra II DNA library prep kit manual). Pipetting precision is very important, especially when using SPRI beads for size selection, and caution and a reliable multichannel pipette (in combination with sterile dispensers) are highly recommended for high‐throughput plate work.

MPCs can be purchased from many verified vendors, and costs vary widely. If 3D printing is available, MPCs can be built from scratch, allowing for extreme control over every aspect of design (models available at, e.g., https://www.thingiverse.com/ or https://www.yeggi.com/). It is important to select the correct type (i.e., neodymium‐grade N42 nickel‐coated) and shape of magnets beforehand to optimize their fit on the custom 3D plate or rack. MPCs designed for plates can assist with high throughput, but we have found that designs where magnets are fixed to the sides of each well (e.g., Promega's MagnaBot) are more efficient than ring‐magnet designs; the latter are typically found in automated pipetting machines (e.g., epMotion [Eppendorf North America, Hauppauge, New York, USA]).

Spin columns are often used for the purification of samples in commercial DNA extraction kits (e.g., QIAquick PCR Purification Kit [QIAGEN], DNA Clean & Concentrator Kits [Zymo Research], or Wizard DNA Clean‐Up System [Promega Corporation]). Traditionally, this method generates a sizeable amount of consumable waste as the columns are single use only. It is possible to intensively sterilize the used columns and replace the filters within (Shi et al., 2018); however, this is more appropriate for low‐throughput studies as it is time‐consuming. Furthermore, the tubes do not facilitate the use of multichannel pipettes. Overall, the spin columns are less expensive than off‐the‐shelf SPRI beads, but more costly than homebrew beads (and less convenient for high‐throughput applications).

## LIBRARY PREPARATION

The preparation of genomic DNA libraries for high‐throughput sequencing is commonly referred to as library prep, and is typically conducted using commercially available kits such as TruSeq Nano, NEBNext Ultra II, or KAPA HyperPrep, although homebrew protocols exist (Rohland and Reich, 2012; Mariac et al., 2014; Ojeda et al., 2019). When samples are multiplexed for sequencing, library prep involves a few general stages: (1) DNA preparation, end blunting, and A‐tailing; (2) ligation of Illumina sequencing adapters (used in sequencing) and index barcodes unique to each multiplexed sample; (3) size selection to reduce fragment size distribution; and (4) PCR amplification of the library (this latter step is not always required and sometimes preferably avoided; PCR‐free library prep kits exist, e.g., TrueSeq and KAPA). All of these steps must be completed before the hybridization stage, in which the specific adapter sequence is prevented from target capture via blockers, and the choice of library prep method may affect target capture efficiency. For example, the myBaits (Arbor Biosciences, Ann Arbor, Michigan, USA) target capture protocol warns against the use of Nextera‐style adapters, although the most recent version of the protocol (version 4.1) describes a step to pre‐treat streptavidin beads to prevent adapters from competing with probes during hybridization.

The library prep stage remains one of the most costly per‐sample steps of the target capture workflow. However, in all of the single‐sample library preps we have tried, costs can be reduced by simply conducting the protocol in reduced volumes: one‐half volumes for Tru‐Seq Nano and NEBNext Ultra II DNA (one‐third volumes are also possible but require ultra‐accurate pipetting), and one‐fourth volumes for KAPA HyperPrep; although the DNA Library Prep Kit (abm, Richmond, British Columbia, Canada) offers a noteworthy competitive price. Reduced volumes extend beyond library kits into adapters (for low starting DNA concentrations, adapters can be diluted in nuclease‐free water to reduce dimer formation) and indexing oligos (preferably dual to overcome index hopping when multiplexing; Kircher et al., 2011). When working at half‐volume, NEBNext Multiplex Oligos for Illumina (dual index primers sets 1 and 2, which come in individual tubes) can easily be combined to simultaneously sequence four plates (e.g., plate 1: i5 1–8 and i7 1–12; plate 2: i5 9–16 and i7 1–12; plate 3: i5 1–8 and i7 13–24; and plate 4: i5 9–16 and i7 1–12); commercial pre‐plated dual index oligos (available from New England Biolabs, NuGEN [TECAN], etc.) restrict the number of possible combinations. Further cost reductions may be achieved by using custom‐made oligonucleotides for indexing such as the Adapterama system (Glenn et al., 2019; https://baddna.uga.edu/adapterama-ordering.html, accessed 1 March 2020), which are compatible with the aforementioned library prep kits for Illumina and provide thousands of unique index combinations. Costs may also be reduced through the use of homebrew SPRI beads (see above), but we urge caution, as homebrew beads may not have the same specificity as the ones provided in library prep kits; as mentioned above, we recommend extensive testing of homebrew beads in the same solute conditions as during library prep, using a DNA ladder and gel.

Further reduction of costs for library prep will require the use of new techniques that allow for multiplexing much earlier in the protocol. Several pooled library prep methods are already available, including PlexWell (seqWell, Boston, Massachusetts, USA) and Swift 2S Turbo (Swift Biosciences, Ann Arbor, Michigan, USA). These recent additions have per‐sample prices comparable to single‐sample preps at a fraction of the time required for bench work. However, because the pooled methods often rely on single‐indexing with Nextera adapters, additional testing is needed to investigate whether the pooled library prep techniques maintain target capture efficiency at lower per‐sample costs.

## HYBRIDIZATION AND ENRICHMENT

RNA probes are preferably used in target capture kits due to the higher hybridization efficiency and stability of RNA‐DNA versus DNA‐DNA unions (Lesnik and Freier, 1995). These RNA probes (e.g., myBaits, SureSelectXT [Agilent Technologies]) should be stored at −80°C, as they are sensitive to freeze‐thaw cycles due to the activities of ribonucleases above −20°C (Ma et al., 2004), but probes can be thawed at least twice without losing efficiency and stability. Researchers lacking −80°C storage may encounter issues using RNA probes and are encouraged to order small batches of chosen RNA‐probe kit once their genomic libraries have been prepared. Alternatively, xGen Lockdown Probe Pools (Integrated DNA Technologies, Coralville, Iowa, USA) can be stored at −20°C (as they are not in suspension). Nonetheless, a few custom kits are available based on DNA probes that do not have these temperature requirements: Twist Custom Panels (Twist Bioscience, San Francisco, California, USA), Nextera Rapid Capture Custom Enrichment Kit (Illumina), SeqCap (Roche), or HaloPlex (Agilent Technologies).

In the myBaits protocol, reagents for liquid phase target enrichment are sold in units of “hybridization reactions” (e.g., eight reactions or 96 reactions). MyBaits kits contain RNA probe sequences, blockers for the chosen library adapter sequences, streptavidin beads, and other reagents for target enrichment of Illumina libraries. Initially, myBaits protocols specified a single specimen per hybridization reaction (https://arborbiosci.com/wp-content/uploads/2018/04/MYbaits-manual-v1.pdf, accessed 1 March 2020), and this is still recommended for very small DNA inputs, such as those from ancient DNA projects. The most recent version of the myBaits protocol (version 4.01, https://arborbiosci.com/wp-content/uploads/2018/04/myBaits-Manual-v4.pdf, accessed 30 September 2019) recommends users explore multiplexing by beginning with four samples, with 125 ng of input library per sample. However, many successful targeted sequencing projects have gone beyond this level of multiplexing; for example, Liu et al. (2019) recovered 150 protein‐coding nuclear genes across mosses (Bryopsida) using 96 libraries multiplexed in a single myBaits reaction. However, pooling strategies using 24–48 libraries per myBaits reaction are more common (e.g., Johnson et al., 2016; Villaverde et al., 2018; Soto Gomez et al., 2019; Zerega and Gardner, 2019).

**Figure 3:**
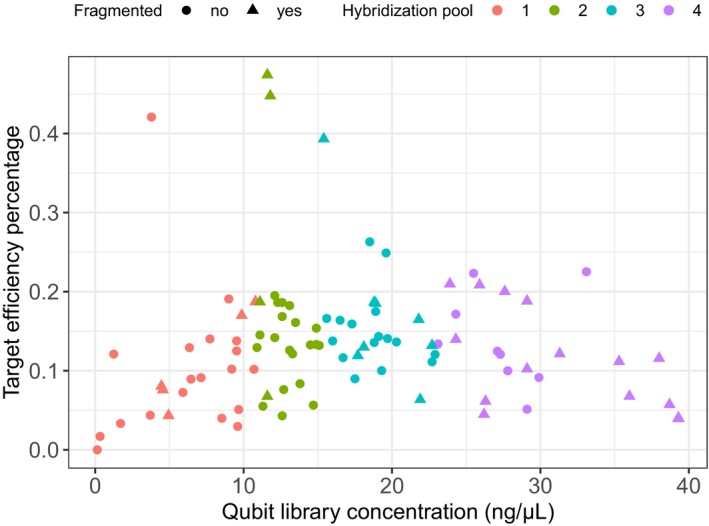
Pooling by concentration for hybridization has a minimal effect on target capture efficiency. The DNA concentration of DNA libraries from 95 samples (plus one negative control) spanning 24 species of angiosperms were tested using a Qubit fluorometer (Thermo Fisher Scientific, Waltham, Massachusetts, USA). Liquid‐phase hybridization with RNA probes (Angiosperms353 version 0.1; Arbor Biosciences, Ann Arbor, Michigan, USA) was conducted in four pools, each containing 24 samples chosen based on their Qubit concentration. Target efficiency is measured as the percentage of sequenced reads mapping to the targeted loci. The four high‐efficiency libraries are from the same species (*Nerisyrenia camporum* Greene, Brassicaceae), which has a close phylogenetic relationship with target sequences in Angiosperms353.

Users considering multiplexing are often cautioned to take careful consideration to produce a pool with equimolar contributions from each library (see myBaits manual). Calculating library molarity requires quantitation of every library for both concentration and size distribution when using fragment analysis methods such as the aforementioned BioAnalyzer and TapeStation, or via qPCR, which would quickly drive up costs. Fortunately, good results can be attained by pooling samples by concentration alone. A random sampling of a few medium‐ to high‐concentration libraries can be checked for fragment distribution, and the median fragment size value can be used to estimate a range of molarities. For example, libraries can be pooled in sets of 24 broadly grouped by concentrations calculated using the Qubit fluorometer (Fig. 3). Using the concentrations, libraries can be added to each pool so that DNA input (in nanograms) from each library within a pool is the same. This pooling strategy method has relatively limited effect on the target enrichment efficiency observed across 96 libraries sequenced using the Angiosperms353 probe set (Fig. 3). It is still recommended to check the molarity of each library pool following hybridization and enrichment to decide how to combine the pools for sequencing.

In the libraries shown in Fig. 3, costs of hybridization were further reduced with a 3 : 1 (water : probe) dilution of RNA probes in nuclease‐free water, while keeping all other myBaits reagents at the specified concentration. This means that 96 libraries can be hybridized using the RNA probes from one myBaits hybridization reaction (as done by Liu et al., 2019); however, this effort can be separated into multiple pools by diluting the RNA probes. Pooling fewer samples per hybridization reaction allows for more flexibility; for example, outgroup samples could be hybridized separately to avoid competition with ingroup samples that are more closely related to the species used to design the probes. For large projects, additional supplies of the undiluted myBaits reagents will be needed, but the overall cost of the myBaits portion of the Hyb‐Seq workflow can be considerably less than US$1 per sample using these modifications.

## SEQUENCING

Choosing the appropriate sequencing platform will depend on the number of samples to be sequenced, the desired level of sequencing depth (coverage), and how important flanking regions and other off‐target sequences are to the project. Sequencing depth will depend directly on the target capture efficiency (i.e., the percentage of reads that map to targeted genes) or, if the Hyb‐Seq approach is used (i.e., mixing genome skimming with enriched libraries in a given percentage), to maximize the recovery of off‐target high‐copy regions. In recent studies, target capture efficiency has varied from 15–25% for universal probe designs, including the Angiosperms353 enrichment panel (Fig. 3) (see also Johnson et al., 2018; Brewer et al., 2019), whereas probes designed for taxon‐specific applications can have efficiencies at 80% or higher (Vatanparast et al., 2018). For sequencing of targeted exons alone, recovery of full‐length target sequences has a logarithmic relationship with sequencing effort (Johnson et al., 2018). For a universal enrichment panel design, such as Angiosperms353, a goal of 300,000 reads per sample is sufficient to recover a large proportion of targeted genes given a 25% enrichment efficiency. To optimize the off‐target fraction, one million reads per sample may be required to ensure coverage for flanking regions and organelles is adequate (Johnson et al., 2018). These guidelines indicate that a single flow cell of Illumina MiSeq (reagent chemistry version 3) can reliably recover sequences for 96 enriched libraries (Fig. 3).

Additional multiplexing is required to further reduce sequencing costs, but this entails using a sequencing platform other than the MiSeq. The Illumina NextSeq (550 and 550Dx) and HiSeq series (2000, 2500, 3000/4000, and X Five and Ten) have much higher read outputs, but the tradeoff is read length. Whereas the MiSeq platform can accommodate paired reads of 300 bp, the longest read length available for the NextSeq and HiSeq platforms is 150 bp. The iSeq and MiniSeq series also produce 150‐bp reads, but output fewer reads than any of the aforementioned platforms, considerably limiting multiplexing capabilities. One of the most cost‐effective per‐sample sequencing platforms for large‐scale multiplexing—e.g., four plates (384 samples) at a time—is the Illumina HiSeq X Ten platform (ca. 440 million 2 × 150 bp paired‐end reads), which additionally has lower levels of index hopping (likely to affect ancient DNA samples) than other Illumina platforms (van der Valk et al., 2019). The main tradeoff is in the reduced recovery of flanking noncoding regions (i.e., introns), which can only be reliably retrieved with the MiSeq version 3 chemistry. In contrast, as target efficiency and multiplexing options increase, the recovery of high‐copy non‐targeted regions, such as transposons or organellar DNA, will only be possible with sequencing outputs greater than that which the MiSeq achieves.

Overall, we would recommend that researchers attempting pilot studies with targeted sequencing utilize the lower per‐sequencing‐run cost of MiSeq (or iSeq and MiniSeq if available) until the desired sequencing output is established. Sequencing using other platforms is also possible but has been less well explored (e.g., Ion Torrent Proton [Thermo Fisher Scientific]; Lesur et al., 2018). Larger projects will be able to take advantage of lower per‐sample costs of the higher‐throughput platforms (e.g., NovaSeq 6000 [Illumina]; Gardiner et al., 2019). In the future, new developments in high‐throughput sequencing with longer read lengths (e.g., NovaSeq S Prime flow cell 2 × 250 bp) or single‐molecule sequencing (e.g., PacBio [Pacific Biosciences, Menlo Park, California, USA], Wolf et al., 2018; MinION [Oxford Nanopore Technologies, Oxford, United Kingdom], Bethune et al., 2019; Li et al., 2019) could facilitate the recovery of flanking regions, but at this time these newer sequencing platforms remain relatively unexplored for target capture projects (but see Gilpatrick et al., 2020; Kovaka et al., 2020; Payne et al., 2020).

## OVERALL COST CONSIDERATIONS

The previous sections illustrated that each stage of the targeted sequence capture workflow has a variety of options for cutting costs in comparison with a conventional target capture workflow (e.g., DNeasy DNA extraction kit, Covaris sonication, and commercially available kits for library preparation and hybridization) by more than 50% (Fig. 1, Table 1). Modifications of this workflow could cut costs even further and will depend on the research question in order to balance considerations including input DNA quality, sequencing depth, phylogenetic scope, number of samples, and equipment availability. Together, these alternatives should allow most research groups to leverage the data generation power of targeted sequencing on a reasonable budget.
